# Biological Warfare at the 1346 Siege of Caffa

**DOI:** 10.3201/eid0809.010536

**Published:** 2002-09

**Authors:** Mark Wheelis

**Affiliations:** * University of California, Davis, California USA

**Keywords:** plague, biological warfare, BW, Caffa, Black Death, de’ Mussi, de Mussis, Kaffa

## Abstract

On the basis of a 14th-century account by the Genoese Gabriele de’ Mussi, the Black Death is widely believed to have reached Europe from the Crimea as the result of a biological warfare attack. This is not only of great historical interest but also relevant to current efforts to evaluate the threat of military or terrorist use of biological weapons. Based on published translations of the de’ Mussi manuscript, other 14th-century accounts of the Black Death, and secondary scholarly literature, I conclude that the claim that biological warfare was used at Caffa is plausible and provides the best explanation of the entry of plague into the city. This theory is consistent with the technology of the times and with contemporary notions of disease causation; however, the entry of plague into Europe from the Crimea likely occurred independent of this event.

The Black Death, which swept through Europe, the Near East, and North Africa in the mid-14th century, was probably the greatest public health disaster in recorded history and one of the most dramatic examples ever of emerging or reemerging disease. Europe lost an estimated one quarter to one third of its population, and the mortality in North Africa and the Near East was comparable. China, India, and the rest of the Far East are commonly believed to have also been severely affected, but little evidence supports that belief (1).

A principal source on the origin of the Black Death is a memoir by the Italian Gabriele de’ Mussi. This memoir has been published several times in its original Latin (2,3) and has recently been translated into English (4) (although brief passages have been previously published in translation, see reference [5]). This narrative contains some startling assertions: that the Mongol army hurled plague-infected cadavers into the besieged Crimean city of Caffa, thereby transmitting the disease to the inhabitants; and that fleeing survivors of the siege spread plague from Caffa to the Mediterranean Basin. If this account is correct, Caffa should be recognized as the site of the most spectacular incident of biological warfare ever, with the Black Death as its disastrous consequence. After analyzing these claims, I have concluded that it is plausible that the biological attack took place as described and was responsible for infecting the inhabitants of Caffa; however, the event was unimportant in the spread of the plague pandemic.

## Origin of the 14th-Century Pandemic

**Figure 1:**
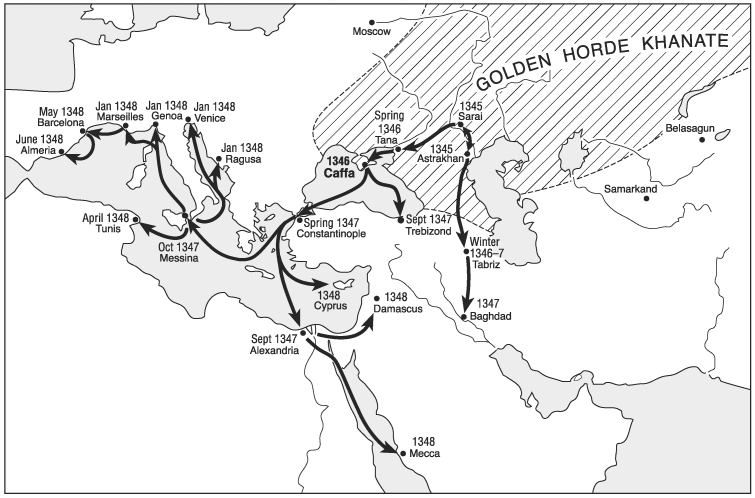
Tentative chronology of the initial spread of plague in the mid-14th century (12–14).

The disease that caused this catastrophic pandemic has, since Hecker (6), generally been considered to have been plague, a zoonotic disease caused by the gram-negative bacterium *Yersinia pestis*, the principal reservoir for which is wild rodents (7–11). The ultimate origin of the Black Death is uncertain—China, Mongolia, India, central Asia, and southern Russia have all been suggested (see Norris [1] for a discussion of the various theories). Known 14th-century sources are of little help; they refer repeatedly to an eastern origin, but none of the reports is first-hand. Historians generally agree that the outbreak moved west out of the steppes north of the Black and Caspian Seas, and its spread through Europe and the Middle East is fairly well documented (Figure 1). However, despite more than a century of speculation about an ultimate origin further east, the requisite scholarship using Chinese and central Asian sources has yet to be done. In any event, the Crimea clearly played a pivotal role as the proximal source from which the Mediterranean Basin was infected.

## Historical Background to the Siege of Caffa

Caffa (now Feodosija, Ukraine) was established by Genoa in 1266 by agreement with the Kahn of the Golden Horde (15). It was the main port for the great Genoese merchant ships (16–20), which connected there to a coastal shipping industry to Tana (now Azov, Russia) on the Don River. Trade along the Don connected Tana to Central Russia, and overland caravan routes linked it to Sarai and thence to the Far East (12,19,20).

Relations between Italian traders and their Mongol hosts were uneasy, and in 1307 Toqtai, Kahn of the Golden Horde, arrested the Italian residents of Sarai, and besieged Caffa. The cause was apparently Toqtai’s displeasure at the Italian trade in Turkic slaves (sold for soldiers to the Mameluke Sultanate). The Genoese resisted for a year, but in 1308 set fire to their city and abandoned it. Relations between the Italians and the Golden Horde remained tense until Toqtai’s death in 1312 (19).

Toqtai’s successor, Özbeg, welcomed the Genoese back, and also ceded land at Tana to the Italians for the expansion of their trading enterprise. By the 1340s, Caffa was again a thriving city, heavily fortified within two concentric walls. The inner wall enclosed 6,000 houses, the outer 11,000. The city’s population was highly cosmopolitan, including Genoese, Venetian, Greeks, Armenians, Jews, Mongols, and Turkic peoples (21).

In 1343 the Mongols under Janibeg (who succeeded Özbeg in 1340) besieged Caffa and the Italian enclave at Tana (12), following a brawl between Italians and Muslims in Tana. The Italian merchants in Tana fled to Caffa (which, by virtue of its location directly on the coast, maintained maritime access despite the siege). The siege of Caffa lasted until February 1344, when it was lifted after an Italian relief force killed 15,000 Mongol troops and destroyed their siege machines (21). Janibeg renewed the siege in 1345 but was again forced to lift it after a year, this time by an epidemic of plague that devastated his forces. The Italians blockaded Mongol ports, forcing Janibeg to negotiate, and in 1347 the Italians were allowed to reestablish their colony in Tana (19).

## Gabriele de’ Mussi

Gabriele de’ Mussi, born circa 1280, practiced as a notary in the town of Piacenza, over the mountains just north of Genoa. Tononi summarizes the little we know of him (3). His practice was active in the years 1300–1349. He is thought to have died in approximately 1356.

Although Henschel (2) thought de’ Mussi was present at the siege of Caffa, Tononi asserts that the Piacenza archives contain deeds signed by de’ Mussi spanning the period 1344 through the first half of 1346. While this does not rule out travel to Caffa in late 1346, textual evidence suggests that he did not. He does not claim to have witnessed any of the Asian events he describes and often uses a passive voice for descriptions. After describing the siege of Caffa, de’Mussi goes on to say, “Now it is time that we passed from east to west to discuss all the things which we ourselves have seen…”

## The Narrative of Gabriele De’ Mussi

**Figure 2:**
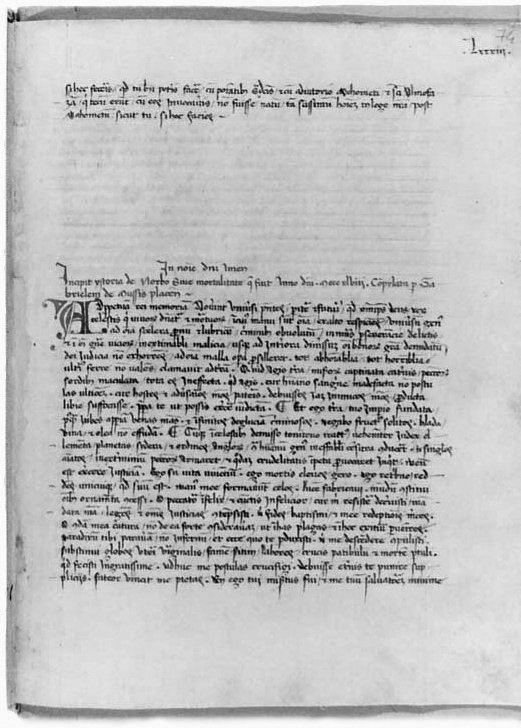
The first page of the narrative of Gabriele de’ Mussi. At the top of the page are the last few lines of the preceding narrative; de’ Mussi’s begins in the middle of the page. The first three lines, and the large “A” are in red ink, as are two other letters and miscellaneous pen-strokes; otherwise it is in black ink. Manuscript R 262, fos 74r; reproduced with the permission of the Library of the University of Wroclaw, Poland.

The de’ Mussi account is presumed to have been written in 1348 or early 1349 because of its immediacy and the narrow time period described. The original is lost, but a copy is included in a compilation of historical and geographic accounts by various authors, dating from approximately 1367 (Figure 2). The account begins with an introductory comment by the scribe who copied the documents: “In the name of God, Amen. Here begins an account of the disease or mortality which occurred in 1348, put together by Gabrielem de Mussis of Piacenza.”

The narrative begins with an apocalyptic speech by God, lamenting the depravity into which humanity has fallen and describing the retribution intended. It goes on:

“…In 1346, in the countries of the East, countless numbers of Tartars and Saracens were struck down by a mysterious illness which brought sudden death. Within these countries broad regions, far-spreading provinces, magnificent kingdoms, cities, towns and settlements, ground down by illness and devoured by dreadful death, were soon stripped of their inhabitants. An eastern settlement under the rule of the Tartars called Tana, which lay to the north of Constantinople and was much frequented by Italian merchants, was totally abandoned after an incident there which led to its being besieged and attacked by hordes of Tartars who gathered in a short space of time. The Christian merchants, who had been driven out by force, were so terrified of the power of the Tartars that, to save themselves and their belongings, they fled in an armed ship to Caffa, a settlement in the same part of the world which had been founded long ago by the Genoese.

“Oh God! See how the heathen Tartar races, pouring together from all sides, suddenly invested the city of Caffa and besieged the trapped Christians there for almost three years. There, hemmed in by an immense army, they could hardly draw breath, although food could be shipped in, which offered them some hope. But behold, the whole army was affected by a disease which overran the Tartars and killed thousands upon thousands every day. It was as though arrows were raining down from heaven to strike and crush the Tartars’ arrogance. All medical advice and attention was useless; the Tartars died as soon as the signs of disease appeared on their bodies: swellings in the armpit or groin caused by coagulating humours, followed by a putrid fever.

 “The dying Tartars, stunned and stupefied by the immensity of the disaster brought about by the disease, and realizing that they had no hope of escape, lost interest in the siege. But they ordered corpses to be placed in catapults^1^ and lobbed into the city in the hope that the intolerable stench would kill everyone inside.^2^ What seemed like mountains of dead were thrown into the city, and the Christians could not hide or flee or escape from them, although they dumped as many of the bodies as they could in the sea. And soon the rotting corpses tainted the air and poisoned the water supply, and the stench was so overwhelming that hardly one in several thousand was in a position to flee the remains of the Tartar army. Moreover one infected man could carry the poison to others, and infect people and places with the disease by look alone. No one knew, or could discover, a means of defense.

“Thus almost everyone who had been in the East, or in the regions to the south and north, fell victim to sudden death after contracting this pestilential disease, as if struck by a lethal arrow which raised a tumor on their bodies. The scale of the mortality and the form which it took persuaded those who lived, weeping and lamenting, through the bitter events of 1346 to 1348—the Chinese, Indians, Persians, Medes, Kurds, Armenians, Cilicians, Georgians, Mesopotamians, Nubians, Ethiopians, Turks, Egyptians, Arabs, Saracens and Greeks (for almost all the East has been affected)—that the last judgement had come.

“…As it happened, among those who escaped from Caffa by boat were a few sailors who had been infected with the poisonous disease. Some boats were bound for Genoa, others went to Venice and to other Christian areas. When the sailors reached these places and mixed with the people there, it was as if they had brought evil spirits with them: every city, every settlement, every place was poisoned by the contagious pestilence, and their inhabitants, both men and women, died suddenly. And when one person had contracted the illness, he poisoned his whole family even as he fell and died, so that those preparing to bury his body were seized by death in the same way. Thus death entered through the windows, and as cities and towns were depopulated their inhabitants mourned their dead neighbours.” (Reproduced with permission from Horrox, pp. 16–20 [4])

The account closes with an extended description of the plague in Piacenza, and a reprise of the apocalyptic vision with which it begins.

## Commentary

In this narrative, de’ Mussi makes two important claims about the siege of Caffa and the Black Death: that plague was transmitted to Europeans by the hurling of diseased cadavers into the besieged city of Caffa and that Italians fleeing from Caffa brought it to the Mediterranean ports.

### Biological Warfare at Caffa

de’ Mussi’s account is probably secondhand and is uncorroborated; however, he seems, in general, to be a reliable source, and as a Piacenzian he would have had access to eyewitnesses of the siege. Several considerations incline me to trust his account: this was probably not the only, nor the first, instance of apparent attempts to transmit disease by hurling biological material into besieged cities; it was within the technical capabilities of besieging armies of the time; and it is consistent with medieval notions of disease causality (22).

Tentatively accepting that the attack took place as described, we can consider two principal hypotheses for the entry of plague into the city: it might, as de’ Mussi asserts, have been transmitted by the hurling of plague cadavers; or it might have entered by rodent-to-rodent transmission from the Mongol encampments into the city.

Diseased cadavers hurled into the city could easily have transmitted plague, as defenders handled the cadavers during disposal. Contact with infected material is a known mechanism of transmission (8–11); for instance, among 284 cases of plague in the United States in 1970–1995 for which a mechanism of transmission could be reasonably inferred, 20% were thought to be by direct contact (24). Such transmission would have been especially likely at Caffa, where cadavers would have been badly mangled by being hurled, and many of the defenders probably had cut or abraded hands from coping with the bombardment. Very large numbers of cadavers were possibly involved, greatly increasing the opportunity for disease transmission. Since disposal of the bodies of victims in a major outbreak of lethal disease is always a problem, the Mongol forces may have used their hurling machines as a solution to their mortuary problem, in which case many thousands of cadavers could have been involved. de’ Mussi’s description of “mountains of dead” might have been quite literally true.

Thus it seems plausible that the events recounted by de’ Mussi could have been an effective means of transmission of plague into the city. The alternative, rodent-to-rodent transmission from the Mongol encampments into the city, is less likely. Besieging forces must have camped at least a kilometer away from the city walls. This distance is necessary to have a healthy margin of safety from arrows and artillery and to provide space for logistical support and other military activities between the encampments and the front lines. Front-line location must have been approximately 250–300 m from the walls; trebuchets are known from modern reconstruction to be capable of hurling 100 kg more than 200 m (25), and historical sources claim 300 m as the working range of large machines (26). Thus, the bulk of rodent nests associated with the besieging armies would have been located a kilometer or more away from the cities, and none would have likely been closer than 250 m. Rats are quite sedentary and rarely venture more than a few tens of meters from their nest (27,28). It is thus unlikely that there was any contact between the rat populations within and outside the walls.

Given the many uncertainties, any conclusion must remain tentative. However, the considerations above suggest that the hurling of plague cadavers might well have occurred as de’ Mussi claimed, and if so, that this biological attack was probably responsible for the transmission of the disease from the besiegers to the besieged. Thus, this early act of biological warfare, if such it were, appears to have been spectacularly successful in producing casualties, although of no strategic importance (the city remained in Italian hands, and the Mongols abandoned the siege).

### Crimea as the Source of European and Near Eastern Plague

There has never been any doubt that plague entered the Mediterranean from the Crimea, following established maritime trade routes. Rat infestations in the holds of cargo ships would have been highly susceptible to the rapid spread of plague, and even if most rats died during the voyage, they would have left abundant hungry fleas that would infect humans unpacking the holds. Shore rats foraging on board recently arrived ships would also become infected, transmitting plague to city rat populations.

Plague appears to have been spread in a stepwise fashion, on many ships rather than on a few (Figure 1), taking over a year to reach Europe from the Crimea. This conclusion seems fairly firm, as the dates for the arrival of plague in Constantinople and more westerly cities are reasonably certain. Thus de’ Mussi was probably mistaken in attributing the Black Death to fleeing survivors of Caffa, who should not have needed more than a few months to return to Italy (16).

Furthermore, a number of other Crimean ports were under Mongol control, making it unlikely that Caffa was the only source of infected ships heading west. And the overland caravan routes to the Middle East from Serai and Astrakhan insured that plague was also spreading south (Figure 1), whence it would have entered Europe in any case. The siege of Caffa, and its gruesome finale, thus are unlikely to have been seriously implicated in the transmission of plague from the Black Sea to Europe.

## Conclusion

Gabriele de’ Mussi’s account of the origin and spread of plague appears to be consistent with most known facts, although mistaken in its claim that plague arrived in Italy directly from the Crimea. His account of biological attack is plausible, consistent with the technology of the time, and it provides the best explanation of disease transmission into besieged Caffa. This thus appears to be one of the first biological attacks recorded (22) and among the most successful of all time.

However, it is unlikely that the attack had a decisive role in the spread of plague to Europe. Much maritime commerce probably continued throughout this period, from other Crimean ports. Overland caravan routes to the Middle East were also unaffected. Thus, refugees from Caffa would most likely have constituted only one of several streams of infected ships and caravans leaving the region. The siege of Caffa, for all of its dramatic appeal, probably had no more than anecdotal importance in the spread of plague, a macabre incident in terrifying times.

Despite its historical unimportance, the siege of Caffa is a powerful reminder of the horrific consequences when disease is successfully used as a weapon. The Japanese use of plague as a weapon in World War II (29) and the huge Soviet stockpiles of *Y. pestis* prepared for use in an all-out war (30) further remind us that plague remains a very real problem for modern arms control, six and a half centuries later (31).

## Footnotes

1 Technically trebuchets, not catapults. Catapults hurl objects by the release of tension on twisted cordage; they are not capable of hurling loads over a few dozen kilograms. Trebuchets are counter-weight-driven hurling machines, very effective for throwing ammunition weighing a hundred kilos or more (22).

2 Medieval society lacked a coherent theory of disease causation. Three notions coexisted in a somewhat contradictory mixture: 1) disease was a divine punishment for individual or collective transgression; 2) disease was the result of "miasma," or the stench of decay; and 3) disease was the result of person-to-person contagion (23).

## References

[1] Norris J. East or West? The geographic origin of the Black Death. Bull Hist Med. 1977;51:1–24. 324542

[2] Henschel AW. Document zur Geschichte des schwarzen Todes. Archives für die gesammte Medizin 1842;2:26–59.

[3] Tononi AG. La Peste Dell’ Anno 1348. Giornale Ligustico de Archeologia. Storia e Letteratura. 1884;11:139–52.

[4] Horrox R, ed. The Black Death. Manchester: Manchester University Press; 1994. p. 14–26.

[5] Derbes VJ. de Mussis and the great plague of 1348. JAMA. 1966;196:179–82. 10.1001/jama.196.1.59 5952188

[6] Hecker JFC. The epidemics of the Middle Ages. London: Sydenham Society; 1844.

[7] Pollitzer R. Plague. Geneva: World Health Organization; 1954.

[8] Benenson AS. Control of communicable diseases manual. Washington: American Public Health Association; 1995.

[9] Bottone EJ. Francisella tularensis, Pasteurella, and *Yersinia pestis*. In: Gorbach SL, Bartlett JG, Blacklow NR, editors. Infectious diseases. Philadelphia: WB Saunders; 1998: p. 1819–24.

[10] Dennis DT, Gratz N, Poland JD, Tikhomirov E. Plague Manual: Epidemiology, distribution, surveillance and control. Geneva: World Health Organization; 1999.

[11] Boyce JM. Yersinia species. In: Mandell GL, Douglas RG Jr, Bennett JE, editors. Principles and practice of infectious disease. 2 ed. New York: John Wiley and Sons; 1985. p.1296–301.

[12] Dols MW. The Black Death in the Middle East. Princeton (NJ): Princeton University Press; 1977.

[13] Gasquet FA. The great pestilence (A. D. 1348–9), now commonly known as the Black Death. London: Simpkin Marshall, Hamilton, Kent & Co.; 1893.

[14] Bartsocas CS. Two fourteenth century Greek descriptions of the "Black Death.”. J Hist Med Allied Sci. 1966;21:394–400. 10.1093/jhmas/XXI.4.394

[15] Vasiliev AA. The Goths in the Crimea. Cambridge (MA): Mediaeval Academy of America; 1936.

[16] Gardiner R. The Age of the galley: Mediterranean oared vessels since pre-classical times. Annapolis (MD): Naval Institute Press; 1995.

[17] Fayle CE. A short history of the world's shipping industry. London: George Allen & Unwin; 1933.

[18] Lewis AR, Runyan TJ. European naval and maritime history, 300–1500. Bloomington (IN): Indiana University Press; 1985.

[19] Grousset R. The empire of the steppes: a history of Central Asia. New Brunswick (NJ): Rutgers University Press; 1970.

[20] Obolensky D. The Byzantine commonwealth: Eastern Europe, 500–1453. London: Weidenfeld and Nicolson; 1971.

[21] Howorth HH. History of the Mongols, from the 9th to the 19th century. New York: Burt Franklin; 1880.

[22] Wheelis M. Biological warfare before 1914. In: Geissler E, Moon JEvC, editors. Biological and toxin weapons: research, development and use from the Middle Ages to1945. London: Oxford University Press; 1999. p. 8–34.

[23] Slack P. Responses to plague in early modern Europe: the implications of public health. In: Mack A, editor. Time of plague; the history and social consequences of lethal epidemic disease. New York: New York University Press; 1991. p. 111–31.

[24] Centers for Disease Control and Prevention. Prevention of plague: recommendations of the Advisory Committee on Immunization Practices (ACIP). MMWR Morb Mortal Wkly Rep. 1996;45:1–15.8531914

[25] Hadingham E. Ready, aim, fire! A risky experiment reveals how medieval engines of war brought down castle walls. Smithsonian. 1975;30:78–87.

[26] Payne-Gallwey R. A summary of the history, construction and effects in warfare of the projectile-throwing engines of the ancients, with a treatise on the structure, power and management of Turkish and other oriental bows of medieval and later times. London: Longmans, Green and Co.; 1907.

[27] Twigg G. The Black Death: a biological reappraisal. London: Batsford Academic and Educational; 1984.

[28] Barnett SA. The rat: a study in behavior. Chicago: University of Chicago; 2000.

[29] Harris SH. Factories of death: Japanese biological warfare 1932–45 and the American cover-up. New York: Routledge; 1994.

[30] Alibek K, Handelman S. Biohazard: The chilling true story of the largest covert biological weapons program in the world—told from the inside by the man who ran it. New York: Random House; 1999.

[31] Inglesby TV, Dennis DT, Henderson DA, Bartlett JG, Ascher MS, Eitzen E, Plague as a biological weapon: medical and public health management. JAMA. 2000;283:2281–90. 10.1001/jama.283.17.2281 10807389

